# Ubiquinol-10 Intake Is Effective in Relieving Mild Fatigue in Healthy Individuals

**DOI:** 10.3390/nu12061640

**Published:** 2020-06-02

**Authors:** Kei Mizuno, Akihiro T. Sasaki, Kyosuke Watanabe, Yasuyoshi Watanabe

**Affiliations:** 1 Osaka City University Center for Health Science Innovation, Osaka 530-0011, Japan; keimizuno@riken.jp (K.M.); akihiro.sasaki@riken.jp (A.T.S.); kyosuke.watanabe@riken.jp (K.W.); 2 RIKEN Compass to Healthy Life Research Complex Program, Kobe, Hyogo 650-0047, Japan; 3 Laboratory for Pathophysiological and Health Science, RIKEN Center for Biosystems Dynamics Research, Kobe, Hyogo 650-0047, Japan; 4 Department of Medical Science on Fatigue, Osaka City University Graduate School of Medicine, Osaka 545-8585, Japan

**Keywords:** ubiquinol, fatigue, oxidative stress, autonomic nerve function, motivation, cognitive function

## Abstract

Our double-blind, placebo-controlled study evaluated effects of ubiquinol, the reduced form of coenzyme Q_10_, on mild fatigue in healthy individuals experiencing fatigue in daily life that had continued for more than 1 and less than 6 months. The participants received 100-mg/day (Ubq100; age 44.0 ± 9.8 years; 14 females and 6 males) or 150-mg/day ubiquinol (Ubq150; age 40.4 ± 11.8 years; 14 females and 8 males) or placebo (Plc; age 41.3 ± 13.4 years; 13 females and 7 males) daily for 12 weeks. Measurements of subjective and objective fatigue were conducted by using questionnaires-based fatigue scales/visual analogue scales and autonomic nerve function/biological oxidation index, respectively, prior to the first dosing and every 4 weeks thereafter. Serum ubiquinol level increased three- to four-fold after 4 weeks and remained significantly higher than that after Plc administration throughout the intake period. Although a higher blood level of ubiquinol was observed with Ubq150 than with Ubq100, the difference was not statistically significant. In both Ubq100 and Ubq150 groups, subjective levels of fatigue sensation and sleepiness after cognitive tasks, which consisted of the modified Advanced Trail Making Test, the modified Stroop Color-Word Test, and the Digit Symbol Substitution Test, improved significantly compared with those in the placebo group, suggesting an anti-fatigue effect. The Ubq150 group demonstrated significant improvement compared with the Plc group regarding subjective level of relaxation after task, sleepiness before and after task, motivation for task, and serum level of oxidative stress. Correlation analysis between blood level of ubiquinol and each evaluated effect suggested a positive relationship with relaxation after task, motivation for cognitive task, and parasympathetic activity. The results of the study suggest that ubiquinol intake relieves mild fatigue in healthy individuals.

## 1. Introduction

Fatigue is a normal sensation that serves to prompt bodily rest following physical or mental exhaustion from daily activities. However, often fatigue is not resolved adequately by rest but is aggravated owing to quality of life (QOL) deterioration. It has been reported that approximately 30% of the Japanese population experience regular subjective fatigue [1,2]. Chronic fatigue syndrome is a disorder diagnosed by profound disabling fatigue that persists for at least 6 months without relief and is not lessened by ordinary rest [3]. It is common in 20 to 50 years old during the prime period of their working life. It has been estimated that it affects some 80,000 to 240,000 individuals in Japan, and approximately one-third of these individuals are bedridden. Prevention of fatigue aggravation is thus of some importance. In the present study, we focused on an intervention study of anti-fatigue effects in healthy individuals experiencing fatigue in daily life that had continued for no longer than the 6 months standpoint from prevention of chronic fatigue.

Fatigue is a condition in which cells and tissues are damaged by reactive oxygen species generated during excessive activity [4]. Recovery from fatigue occurs when this damage is adequately repaired; however, if the damage continues with inadequate repair due to the lack of necessary energy for recovery, an inflammatory response is elicited; this leads to conditions such as malaise and fever, consequently resulting in chronic fatigue [4]. Accordingly, antioxidants that inhibit damage from oxidative stress, and substances that can stimulate energy production are effective against fatigue.

Ubiquinol, a reduced form of coenzyme Q_10_, is present in food and has been reported to have anti-fatigue activity [5]. This substance is a vitamin-like compound used in many countries as an ingredient in foods and supplements. As antioxidants increase ATP production by stimulating mitochondrial electron transport, their bioactivity consists of two elements—antioxidant activity and energy production that both generate an anti-fatigue effect. These two mechanisms likely contributed to many other effects that have been reported in human clinical studies, including enhanced cardiac function, anti-inflammatory effect on hematopoietic cells, sialagogic effects in middle- or advance-aged healthy individuals with dry-mouth condition, and improved conditions of patients with Parkinson’s disease when used in combination with L-dopa [6].

Although ubiquinol is biosynthesized in humans, it is also present in food. Its level in various organs has been reported to reduce with age, suggesting reduction of biosynthetic activity with aging [7]. The biosynthesis pathway of ubiquinol, like that of cholesterol, involves metabolism by hydroxymethylglutaryl-CoA (HMG-CoA) reductase. This is likely the reason why its level in the blood has been reported to decrease by statins that lower the blood-cholesterol level [8]. It has been estimated that 3−5 mg of ubiquinol is ingested daily through diet; this amount is contributed largely by meats and seafood. It has been suggested that the amount of meat intake influences the amount of ubiquinol in the body [9]. According to research in patients with complete nutritional independence, approximately half of the blood ubiquinol is believed to originate from the diet [10]. After absorption, ubiquinol stays in blood as a lipoprotein component. It has been reported that the ubiquinol amount in blood is significantly higher in healthy individuals of advanced age than in elderly bedridden individuals, and that the risk of dementia is reduced approximately by half in individuals having high blood levels of ubiquinol [11].

In an open-label study on ubiquinol intake in patients with chronic fatigue syndrome, cognitive performance indicator of fatigue as well as the questionnaire score of depression symptoms improved along with an increased blood level of ubiquinol [5]. A double-blind study found that the blood ubiquinol level in patients with chronic fatigue syndrome was low prior to ubiquinol intake. Fatigue improvement, reduction in the episodes of wakefulness during sleep, and inhibition of decline in autonomic nerve functions were demonstrated following ubiquinol intake [5]. Another double-blind study in healthy individuals aged approximately 60 years found improvement in the mental QOL including subjective fatigue and improved spontaneous activity [12]. An interventional prospective cohort study among elderly residents suggested beneficial effects on QOL, including subjective fatigue, and improved maintenance of cognitive function [13]. This study also demonstrated that long-term ubiquinol intake over 5 years posed no safety issues. Thus, the beneficial effects of ubiquinol on fatigue have been shown not only in patients with chronic fatigue syndrome but also in healthy individuals; however, data in young individuals are scarce, being limited to a report of beneficial psychological effects and improved athletic performance in female university athletes [14]. Therefore, we targeted a wide range of age in young and old adults in the present study.

The present double-blind study examined the anti-fatigue effect of ubiquinol in healthy participants aged between 20 and 64 years, experiencing mild fatigue in daily life.

## 2. Materials and Methods

### 2.1. Clinical Study

The clinical study employed a written questionnaire on parameters such as fatigue, sleep, and lifestyle habits as well as the autonomic nerve function screening to select participants for evaluation of the anti-fatigue effects of a soft-capsule formulation containing ubiquinol. Seventy-one participants were selected out of 104 healthy individuals experiencing fatigue in daily life that had continued for more than 1 and less than 6 months and who were judged by the principal investigator as not having chronic fatigue syndrome with the checklist of diagnostic criteria [3]. This was a randomized, comparative, placebo-controlled, double-blind, parallel-group study designed in accordance with the recommendations of the Consolidated Standards of Reporting Trials guidelines. Written consent was obtained from participants after they were informed in advance about the study’s objectives, methods, and expected clinical advantages and disadvantages of the treatment. This study was performed in compliance with the principles of the Declaration of Helsinki and was approved by the ethical-review board of the Ethics Committee of Osaka City University Center for Health Science Innovation (OCU-CHSI-IRB no. 16). The study was registered with the UMIN Clinical Trials Registry.

The inclusion criterion required that participants were (1) healthy individuals at least 20 years old and (2) experiencing fatigue in daily life that had continued for more than 1 and less than 6 months. The study exclusion criteria included participants (1) receiving continuous treatment for disease or on medications; (2) intaking coenzyme Q_10_ continuously; (3) with a history of cardiovascular or neurological diseases; (4) with allergies, sleep disorders, or organic diseases that clearly contributed to fatigue; (5) who were pregnant, planning pregnancy, or nursing; (6) who had psychiatric diseases or history thereof; (7) who consumed alcohol in excess or could not avoid the use of medications that could potential affect the outcomes; (8) who had one or more severe diseases, such as diabetes mellitus, liver disease, kidney disease, heart disease, or other diseases; (9) who were participating in another clinical study within 1 month prior to giving consent or were participating in any other clinical trial; and (10) who were judged unsuitable by the principal investigator for any other reasons.

Participants selected through preliminary screening were randomized by age and gender into three groups to receive 100 mg of ubiquinol, 150 mg of ubiquinol, or placebo (capsule without ubiquinol). Four participants who dropped out after the initiation of the study were excluded from analysis as well as five participants who were affected during the course of the study by bone fracture, initiation of treatment for hyperlipidemia, medication use for headache, strenuous exercise (long-distance running), and low rate of trial dietary product intake (≤80%). These screenings were performed based on the result of a 4-weeks daily journal recorded by each participant. Questions about dietary intake and alcohol intake were also included in this daily journal. To judge the subjects for analysis, we carefully checked the daily journal every four weeks, on the 2nd, and 3rd experimental days. The final analysis included 20 participants in the placebo group (age 41.3 ± 13.4 years; 13 females and 7 males), 20 in the 100-mg group (age 44.0 ± 9.8 years; 14 females and 6 males), and 22 in the 150-mg group (age 40.4 ± 11.8 years; 14 females and 8 males).

The trial dietary product was a soft-capsule formula containing 50 mg of ubiquinol. The additional ingredients in capsules were rapeseed oil, diglycerol monooleate, beeswax, soy lecithin, and caramel as a coloring agent. Rapeseed oil was added to the placebo capsule instead of ubiquinol. Participants took three capsules once daily after breakfast continuously for 12 weeks (the placebo group took three placebo capsules; the 100-mg group took two active capsules and one placebo capsule; and the 150-mg group took three active capsules).

The main outcome measures that were evaluated every 4 weeks included the autonomic nerve function, oxidative stress/antioxidative activity of blood samples, reaction time and rate of correct response in cognitive function tasks, subjective fatigue sensation, and the serum ubiquinol level.

### 2.2. Questionnaires

The Chalder Fatigue Scale [15] and a modified version of the Osaka City University Hospital Fatigue Scale [16] were used for evaluating the severity of fatigue. The K6 scale [17] can measure mood and anxiety and the Center for Epidemiological Studies Depression Scale [18] can evaluate the symptoms of depression. The Pittsburgh Sleep Quality Index [19] and the Epworth Sleepiness Scale [20] were used to measure general sleepiness and daytime sleepiness, respectively. Previous studies have confirmed the reliability and validity of the Japanese versions of these questionnaires [16,21,22,23,24,25]. We evaluated the level of subjective fatigue sensation using a visual analogue scale (VAS) from 0 representing “no fatigue,” to 100 representing “total exhaustion” immediately prior to and after cognitive function testing. A VAS for sleepiness, depression, relaxation, and motivation was also used at the same measurement points.

### 2.3. Autonomic Nerve Function

Autonomic nerve function is an objective marker for evaluating fatigue [4]. Data were generated by simultaneous electrocardiography and photoplethysmography using a Vital Monitor 302 system (Fatigue Science Laboratory, Osaka, Japan) in participants sitting quietly with their eyes closed for 3 min. Frequency analyses for R-R interval variation from electrocardiography and a-a interval variation as the second derivative of photoplethysmography were measured. The maximum entropy method that is capable of estimating the power-spectrum density from short time-series data and is adequate for examining short-duration changes in heart-rate variability under different conditions was used [26,27]. Low frequency (LF) was taken as the power within a frequency range of 0.04–0.15 Hz and high frequency (HF) was as being within a frequency range of 0.15–0.4 Hz. HF is vagally mediated [28,29], whereas LF originates from a variety of sympathetic and vagal mechanisms [30,31]. We used log-transformed (ln) LF, ln HF, and ln LF/HF ratio [32] in the present study. Prior to 3-min autonomic nerve function testing, a practice test was conducted for a period of 1 min in accordance with previous studies [32,33,34,35].

### 2.4. Analyses of Oxidative Stress and Antioxidant Activity

The oxidative activity of serum samples was determined using a test for reactive oxygen metabolites-derived compounds (d-ROMs; Diacron International, Grosseto, Italy). The antioxidative activity was determined by measuring the biological antioxidant potential (BAP) (Diacron International) using a JCABM1650 automated analyzer (JEOL, Tokyo, Japan) [36]. The d-ROMs value was expressed in Carratelli Units (1 CARR U = 0.08 mg hydrogen peroxide/dL) [37]. The oxidative stress index (OSI) was calculated using the following formula: OSI = C × (d-ROMs/BAP) where C denotes a coefficient for standardization to set the mean OSI in healthy individuals at 1.0 (C = 8.85) [36]. All serum samples were stored at −80 °C until analysis. Assays for serum d-ROMs and BAP were performed at Yamaguchi University Graduate School of Medicine.

### 2.5. Analysis of Serum Ubiquinol

Serum concentrations of ubiquinol were measured using LC/MS/MS (commissioned at Kaneka Techno Research Corporation). Specifically, 0.1 mL of serum was fractionated immediately after collection, mixed with 0.7 mL of isopropanol to prevent oxidation of ubiquinol and stored at −80°C until measurement. At the time of measurement, samples were centrifuged at 12,000 rpm for 5 min; supernatants were filtered through a membrane filter and subjected to LC/MS/MS analysis using the AB SCIEX Triple Quad5500 instrument.

### 2.6. Cognitive Functions

#### 2.6.1. Modified Advanced Trail Making Test

To assess the cognitive function and volition of subjects, Task E in the modified Advanced Trail Making Test (mATMT) was implemented [38,39,40]. The mATMT is designed to assess cognitive function in terms of the response time and number of correct responses to tasks displayed on a computer screen; it further determines the task-to-task shifting speed as an indicator of volition. It is also used to assess the performance of fatigued humans [41,42]. The participants were asked to respond to a total of 25 visual stimuli (numerals 1–13 and 12 different hiragana characters) displayed on the screen in random positions as follows: participants moved the cursor and clicked on the numerals and hiragana alternatively in the specified order, using the mouse, as quickly as possible for 5 consecutive minutes. During this task, cognitive function was evaluated by the number of correct/erroneous responses. Motivational response was evaluated in terms of response time from the end of one task to the start of the next task, with motivation rated higher as response time shortened. As in our previous studies [35,42], participants practiced for a period of 1 min before performing Task E of the mATMT on each experimental day.

#### 2.6.2. Modified Stroop Color-Word Test

For the second assessment of cognitive function, the modified Stroop Color-Word Test (mSCWT) was used. In this test, the ability of the subject correctly to judge colors and characters in a pedestrian signal displayed on a computer screen was assessed [43]. Participants were asked to perform Task 1 (3 min) and Task 2 (6 min) as quickly and accurately as possible. In Task 1, the subject right-clicked the mouse upon receiving a flickering blue signal and left-clicked upon receiving a flickering red signal. In Task 2, the subject observed and judged characters appearing on the screen. Specifically, the subject right-clicked the mouse only when the word (in Japanese) appeared in blue. In other cases, the mouse was left-clicked. During these tasks, the time elapsed since clicking was displayed in 100-ms increments. Although the colors and characters were displayed randomly in each task, their frequencies were always constant. The frequencies in the Stroop trial (colors and characters mismatched) and the non-Stroop trial (colors and characters matched) were also constant. The result of each cognitive task in terms of it being a correct or error response was continuously presented on the personal computer display. Participants practiced for a total of 3 min (Task 1 for 1 min and Task 2 for 2 min) before performing the cognitive function task [43].

#### 2.6.3. Digit Symbol Substitution Test

The Digit Symbol Substitution Test (DSST) is used to evaluate the visual processing speed; it was implemented as a measure of cognitive function [44,45,46]. Participants performed the task of entering as many numbers corresponding to the signals as possible within the 2-min time limit; the number of correct responses was used as the score.

### 2.7. Actigraphy (Sleep Parameters)

Actigraphy has been established as a valid and objective method of assessing sleep–wake parameters in natural settings [47]. Actigraphy devices are typically worn on the wrist and record movements that can be used to estimate sleep parameters [48]. Actigraphy data were obtained using the Actiwatch Spectrum (Philips Respironics, Murrysville, PA, USA) and processed using Actiware software (version 6.0.6, Philips Respironics, Murrysville, PA, USA); default settings (wake threshold medium, 10 min immobile for both sleep onset and offset criteria) were used. Averaged parameters of sleep time and sleep efficiency were calculated during the 12-week study period.

### 2.8. Statistical Analysis

Each measured value was represented as the mean ± standard deviation. Data analyses were performed by two-way analysis of variance with post-hoc Dunnet’s multiple comparison test. *p*-values were corrected for number of post-hoc tests. Pearson’s correlation analyses were also performed. All *p*-values < 0.05 were considered statistically significant. Statistical analyses were performed using IBM SPSS Statistical Package version 25.0 (IBM, Armonk, NY, USA).

## 3. Results

Table 1 shows the demographic characteristics of the study participants. The participants were randomized into three groups and had similar male to female ratio, mean age, and age range. The 62 analyzed participants were of 20–64 years of age, consisting of 10 participants in their 20s, 16 in their 30s, 15 in their 40s, 18 in their 50s, and 3 in their 60s. The effect of change in temperature was minimized by conducting the study during a relatively warm climate between March and June in Japan. The days and times for tests were fixed as much as possible to prevent the lifestyle pattern of the participants influencing the outcome. However, since the participants who were evaluated on Sunday had a day of rest, we focused the analyses mainly on the degree of change in each outcome measure (∆value).

Before the first dosing, no statistically significant difference was observed among the three groups in any of the parameters. Because we recognized that the standard deviations tended to be large overall, we also analyzed the degree of change from before supplementation considering variation between individuals. Items in which a statistically significant improvement was observed are summarized in Table 2. In VAS for subjective sensation, the 150-mg group differed significantly from the placebo group in scores of VAS for fatigue, sleepiness and relaxation after the cognitive function task, and score of VAS for difference in sleepiness before and after the cognitive function task. Further, motivation in regard to cognitive function task and d-ROMs, serum oxidative stress markers, and ∆ln(LF/HF) balance of autonomic nerve function improved significantly compared with that in the placebo group. In the 100-mg group, scores of VAS for fatigue and sleepiness after the cognitive function task were significantly different compared with those in the placebo group.

**Figure 1:**
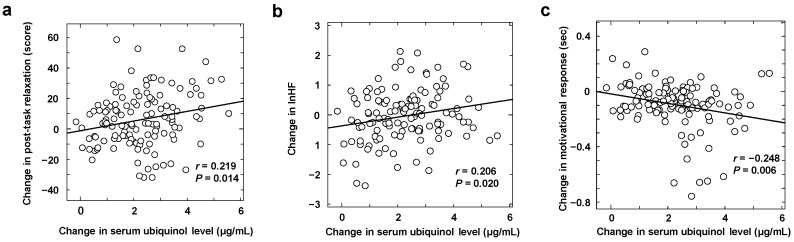
Correlation with change in serum ubiquinol concentration. Visual analogue scale-based post-task relaxation (**a**), log-transformed high-frequency component power (lnHF) (**b**), and motivational response of cognitive task (**c**).

The serum ubiquinol level prior to supplementation was 0.96 ± 0.23 µg/mL (range, 0.50–1.46 µg/mL) and no correlation with age was observed at a level of 1.04 ± 0.22 µg/mL in males and 0.92 ± 0.24 µg/mL in females, although slightly lower levels in females were found (*p* = 0.07). At 4 weeks after the first dose, the groups receiving the active capsule showed a significant increase in serum ubiquinol level both compared with the group receiving placebo and that before first dosing (*p* < 0.001). Although the 150-mg group showed higher levels than the 100-mg group, the difference was not statistically significant. Correlation analysis of the degree of increase in blood ubiquinol level and scores on evaluated items suggested the positive correlation with ∆VAS score for relaxation after cognitive function task (Figure 1a) and ∆lnHF, indicating parasympathetic nerve activity (Figure 1b), and a negative correlation with decline in ∆motivational response of the mATMT (Figure 1c).

The DSST score is inversely related to age. The rise in the DSST score was greater in the ubiquinol-intake groups than in the placebo group; however, the difference was not statistically significant. In the placebo group, a significant increase in the scores was observed at 8 weeks and 12 weeks compared with that before first dosing, suggesting that habituation due to testing every 4 weeks had an influence on learning effect (Table 2). The biochemical parameters did not reveal any changes indicative of safety issues.

## 4. Discussion

In the present double-blind study, we evaluated the anti-fatigue effects of ubiquinol in healthy individuals having mild subjective fatigue. Participants took three capsules of ubiquinol once daily for 12 weeks (the placebo group; 100-mg group or 150-mg ubiquinol groups). Our results indicate a certain level of improvement effects in the oxidative stress, cognitive function, and subjective fatigue sensation and sleepiness.

Fatigue is a condition in which cells and tissues are damaged by reactive oxygen species generated during excessive activity [4]. In our previous study using a complex fatigue animal model where rats are exposed to relatively long-lasting stress and partial sleep deprivation, which humans often experience in their daily lives, plasma levels of total nitric oxide metabolite species were increased, indicating enhancement of systemic oxidative stress [49]. An increase in lipid peroxidation and a decrease in levels of antioxidant enzymes in the brain in the mouse model of chronic fatigue syndrome were also reported [50]. In human studies, the impact of oxidative stress at different levels of fatigue in healthy individuals and patients with chronic fatigue syndrome has been reported [51], suggesting that antioxidants including ubiquinol are effective against fatigue [4,5]. Ubiquinol is an essential electron carrier and proton translocator in the mitochondrial respiratory chain. Ubiquinol is also an obligatory cofactor of the dihydroorotate dehydrogenase [52] and serves as a potent antioxidant in membranes by directly scavenging radicals and regenerating α-tocopherol [53,54]. In the d-ROMs test, oxidative stress in the blood was evaluated by measuring hydroperoxides serum metabolites resulting from oxidative stress. In the serum, ubiquinol is primarily incorporated in lipoproteins, such as low-density lipoprotein (LDL), and its reducing activity on LDL-lipid peroxides and α-tocopherol radicals has been reported [55]. The significant reduction in d-ROMs level after ubiquinol intake in the 150-mg group is thought to be a result of an increased antioxidant ubiquinol level in the blood. In the 100-mg group, the significant reduction in d-ROMs level by ubiquinol administration was not observed influenced by some outliers of d-ROMs, which is above the second positive standard deviation (+2 SD) of mean value in the placebo group.

The participants were assessed every 4 weeks on pre-fixed Saturday or Sunday mornings. The subjective fatigue sensation was evaluated with VAS before and after cognitive function tasks, which was also related to fatigue load. Although fatigue load includes both exercise load, such as bicycle pedaling, and mental load that imitate work on a computer, mental fatigue tasks were implemented in this study. Ubiquinol intake led to significant improvements in subjective fatigue sensation and sleepiness after the cognitive-fatigue load in both the 100-mg and 150-mg groups compared with that in the placebo group. These results appear to support previous reports of beneficial effects of ubiquinol on fatigue in the daily life of individuals of middle and advanced ages [12].

After 4 weeks of 150-mg ubiquinol intake, the motivated response to cognitive function tasks became faster and the cognitive function improved. No significant difference from the placebo group was observed after 8 and 12 weeks of intake; this suggests that habituation to the task had an impact on the placebo group whose response times gradually shortened, decreasing every to −0.03 s after 4 weeks, −0.08 s after 8 weeks, and −0.10 s after 12 weeks compared with the values before the first dose. The 150-mg group showed consistency at −0.14 s after 4 weeks, −0.13 s after 8 weeks, and −0.14 s after 12 weeks, suggesting improvement and maintenance of cognitive functions. Previous studies have also reported improvements in cognitive function following ubiquinol intake in patients with chronic fatigue syndrome [5] as well as in individuals of an advanced age [56].

The motivated response to cognitive function tasks (mATMT) and antioxidative activity also improved in a 4-week study in healthy individuals following the intake of hydrogen-rich water having antioxidative functions [35]. We believe that the correction of mitochondrial function and oxidative stress due to ubiquinol intake also took place in our study and was responsible for improved cognitive function performance; cognitive performance improvement correlated blood ubiquinol level. Conversely, no significant improvement due to ubiquinol intake was observed in terms of the DSST scores. DSST is a cognitive challenge in which participants quickly and accurately enter combinations of figures and numbers, and scores are reported to decrease significantly in patients with dementia or depression [44]. An interventional prospective cohort study with ubiquinol showed that the DSST scores correlated inversely with age and that the scores improved following ubiquinol intake for half a year or longer, suggesting beneficial effects in terms of improvement and maintenance of cognitive functions [13]. In the present study, the placebo group showed a gradual increase in the score from before intake, and scores at 8 and 12 weeks were significantly higher than that before the first dose. This is thought to have resulted from increased DSST proficiency from performing the test every 4 weeks. In the ubiquinol groups, compared with the scores before the first dose, scores increased significantly starting at 4 weeks of intake, and in the 150-mg group, it was also significant compared with the placebo group. However, no significant difference was observed between the degrees of change in each group. Although the effect of ubiquinol on DSST score improvement was not clear in this study, this could be owing to the relatively young age of the participants and the short intake period of 3 months.

In the present study, an improvement in autonomic nerve function was observed in the 150-mg group only after 4 weeks of intake, and no significant improvement was observed after 8 or 12 weeks. However, the parasympathetic indicator lnHF and VAS score for relaxation after the cognitive function task correlated positively with the blood ubiquinol level. It was clear that continuous intake of ubiquinol also results in increased parasympathetic activity in patients with chronic fatigue syndrome [5]. Furthermore, symptoms of depression have been reported to improve along with the blood ubiquinol level in patients with chronic fatigue syndrome [5]. It may be inferred from the results of the present study that antioxidative activity due to ubiquinol intake modulated negative emotions and not only provided a subjective sense of relaxation but also stimulated the parasympathetic activity.

The improvements in fatigue, relaxation, cognitive function, and autonomic nerve function obtained in this study from ubiquinol intake suggest a relationship with improved central nervous system functions. Molecular brain imaging and brain-activity-imaging studies based on positron emission tomography (PET) and functional magnetic resonance imaging (fMRI) have shown the specific regions of the brain that are associated with central management of these functions and sensations. The orbitofrontal area is responsible for managing information related to subjective fatigue sensation [57]; the corpus striatum is involved in management of motivated response during the performance of cognitive tasks [58]; and the anterior cingulate gyrus is associated with control of the autonomic nerve activity [59]. Since it has been considered that fatigue could potentially accelerate the oxidation of the central nervous system including these regions of the brain [60], the present results may suggest that ubiquinol intake may have inhibited intracerebral oxidation. Interestingly, when intracerebral uptake of ubiquinol and ubiquinone were verified using PET, the uptake level of ubiquinol was considerably higher than that of ubiquinone [61]. Therefore, improvements in fatigue, relaxation, cognitive function, and autonomic nerve function seen in the present study might also be greater with ubiquinol than with ubiquinone. This point could be clarified by conducting comparative clinical trials of ubiquinone versus ubiquinol.

There are limitations in the present study. In order to generalize our results, further study involving a larger number of participants is essential. We did not evaluate the effect of energy production by ubiquinol administration in the present study. Recovery from fatigue occurs when cells and tissues damaged by reactive oxygen species have been adequately repaired. However, the damage continues with inadequate repair in the case of the lack of necessary energy for recovery [4]. We previously demonstrated not only enhancement of oxidative stress but also decreases in amount of ATP and metabolites involved in the tricarboxylic acid (TCA) cycle, such as cis-aconitate and isocitrate, in the complex fatigue animal model [49]. Likewise, in patients with chronic fatigue syndrome, we revealed a profile of abnormal energy metabolism resulting from deficiencies in aconitase activity in the TCA cycle [62]. Accordingly, ubiquinol that avoids damage from oxidative stress and that can stimulate energy production may be effective against fatigue. Further study using metabolome analysis is needed to confirm the effect of energy production by ubiquinol administration on healthy individuals with mild fatigue. In the present study, although we mainly examined the effects of ubiquinol on the central nervous system, we did not directly evaluate the dynamics of ubiquinol in the brain. Human neuroimaging studies using PET and fMRI are thus underway in our laboratory to identify the mechanisms underlying the effects of ubiquinol intake on the central nervous system that can attenuate fatigue.

## 5. Conclusions

In the present study, results of evaluation of the effects of ubiquinol in healthy individuals with mild fatigue suggested that ubiquinol contributes to the improvement of QOL in individuals with mild fatigue by reducing fatigue and sleepiness following cognitive function load, promoting motivated engagement with cognitive function tasks while providing a relaxing effect, and reducing oxidative stress.

## Figures and Tables

**Table 1:** Participant characteristics.

Variables	Placebo	100 mg/day	150 mg/day
Number	20	20	22
Sex (male:female)	7:13	6:14	8:14
Age (years; mean ± S.D.)	41.3 ± 13.4	44.0 ± 9.8	40.4 ± 11.8
Age (range in years)	20–64	23–58	20–61

S.D.: standard deviation.

**Table 2:** Effects of ubiquinol by dosage group.

Item	Week	Placebo	100 mg	150 mg
Serum ubiquinol (µg/mL)	0	0.96 ± 0.19	1.00 ± 0.29	0.93 ± 0.20
	4	0.82 ± 0.19	2.96 ± 1.23 ***	3.53 ± 1.01 ***
	8	1.15 ± 0.44	3.29 ± 1.40 ***	3.93 ± 1.40 ***
	12	0.76 ± 0.17	2.61 ± 1.07 ***	3.27 ± 1.20 ***
Pre-task fatigue (score)	0	-	-	-
	4	4.7 ± 22.2	−5.1 ± 16.5	−4.1 ± 16.9
	8	7.0 ± 29.3	−4.5 ± 22.5	−3.4 ± 22.6
	12	0.9 ± 22.0	−5.4 ± 20.7	−1.9 ± 22.1
Post-task fatigue (score)	0	-	-	-
	4	5.4 ± 28.1	−14.1 ± 11.9 **	−9.5 ± 21.8 *
	8	3.7 ± 23.0	−7.3 ± 19.5	−12.3 ± 21.4 *
	12	−2.1 ± 25.9	−10.9 ± 17.7	−3.6 ± 20.8
Post-task sleepiness (score)	0	-	-	-
	4	11.9 ± 27.0	−0.8 ± 13.5 *	−7.6 ± 18.7 **
	8	4.8 ± 25.1	−3.6 ± 20.4	−7.2 ± 25.4
	12	3.8 ± 28.2	−2.2 ± 19.4	−3.3 ± 24.7
Post-task mood reduction (score)	0	-	-	-
	4	5.8 ± 14.0	−8.5 ± 16.2 *	0 ± 26.4
	8	2.2 ± 13.6	−4.4 ± 23.5	−1.6 ± 23.9
	12	2.8 ± 15.6	−10.8 ± 16.8 *	4.1 ± 24.4
ESS: sleepiness (score)	0	-	-	-
	4	0.65 ± 3.98	−1.10 ± 4.95	−0.59 ± 3.94
	8	1.00 ± 4.95	−1.47 ± 2.74 *	−0.18 ± 3.80
	12	0.50 ± 3.81	−0.32 ± 3.25	−0.86 ± 4.61
Post-task relaxation (score)	0	-	-	-
	4	4.5 ± 16.9	3.0 ± 16.7	5.3 ± 18.0
	8	−0.3 ± 14.1	4.2 ± 18.2	11.7 ± 20.3 *
	12	0.6 ± 17.2	4.6 ± 13.2	7.0 ± 17.6
Sleepiness during task (score)	0	-	-	-
	4	6.1 ± 16.1	1.6 ± 18.5	−8.5 ± 18.3 *
	8	−5.5 ± 14.7	−1.7 ± 22.2	−7.0 ± 20.2
	12	0.9 ± 15.7	−0.2 ± 22.1	−9.3 ± 27.3
Motivational response (sec)	0	-	-	-
	4	−0.03 ± 0.11	−0.03 ± 0.11	−0.14 ± 0.21 *
	8	−0.08 ± 0.11	−0.08 ± 0.11	−0.13 ± 0.17
	12	−0.10 ± 0.11	−0.04 ± 0.11	−0.14 ± 0.20
d-ROMs (U.CARR)	0	-	-	-
	4	−17.3 ± 23.8	−1.0 ± 34.9	−26.4 ± 35.8
	8	−7.3 ± 30.4	−6.1 ± 34.9	−31.0 ± 42.3 *
	12	−19.6 ± 33.9	−8.0 ± 47.9	−29.4 ± 41.5
DSST (score: absolute value)	0	63.6 ± 9.37	60.7 ± 9.07	68.4 ± 11.6
	4	65.4 ± 9.86	63.9 ± 8.54	72.1 ± 13.3 *
	8	67.1 ± 8.56	65.0 ± 9.24	73.9 ± 13.8 *
	12	69.9 ± 9.22	66.0 ± 11.5	76.0 ± 13.8
ln(LF/HF) (ratio)	0	-	-	-
	4	0.26 ± 0.72	0.13 ± 0.72	0.28 ± 1.04
	8	0.47 ± 0.82	0.03 ± 0.95	−0.02 ± 0.84 *
	12	0.41 ± 0.81	0.36 ± 0.85	0.32 ± 1.15

Data show means ± standard deviations. * *p* < 0.05, ** *p* < 0.01, *** *p* < 0.001. Dunnett’s multiple comparison vs. placebo. ESS: Epworth Sleepiness Scale, d-ROMs: reactive oxygen metabolites-derived compounds, DSST: Digit Symbol Substitution Test, ln(LF/HF): log-transformed low-frequency component power/high-frequency component power.

## References

[1] Kuratsune H. (2007). Overview of chronic fatigue syndrome focusing on prevalence and diagnostic criteria. Nihon Rinsho.

[2] Kuratsune H., Kondoh K., Ikuta K., Yamanishi K., Watanabe Y., Kitani T. (2001). Chronic fatigue syndrome (CFS). Nihon Naika Gakkai Zasshi.

[3] Fukuda K., Straus S.E., Hickie I., Sharpe M.C., Dobbins J.G., Komaroff A., International Chronic Fatigue Syndrome Study Group (1994). The chronic fatigue syndrome: A comprehensive approach to its definition and study. Ann. Intern. Med..

[4] Watanabe Y., Kuratsune H., Kajimoto O. (2012). Biochemical indices of fatigue for anti-fatigue strategies and products. The Handbook of Operator Fatigue.

[5] Fukuda S., Nojima J., Kajimoto O., Yamaguti K., Nakatomi Y., Kuratsune H., Watanabe Y. (2016). Ubiquinol-10 supplementation improves autonomic nervous function and cognitive function in chronic fatigue syndrome. Biofactors.

[6] Yoritaka A., Kawajiri S., Yamamoto Y., Nakahara T., Ando M., Hashimoto K., Nagase M., Saito Y., Hattori N. (2015). Randomized, double-blind, placebo-controlled pilot trial of reduced coenzyme Q10 for Parkinson’s disease. Parkinsonism Relat. Disord..

[7] Kalen A., Appelkvist E.L., Dallner G. (1989). Age-related changes in the lipid compositions of Rat and Human tissues. Lipids.

[8] Mabuchi H., Higashikata T., Kawashiri M., Katsuda S., Mizuno M., Nohara A., Inazu A., Koizumi J., Kobayashi J. (2005). Reduction of serum ubiquinol-10 and ubiquinone-10 levels by atrobastatin in hypercholesterolemic patients. J. Atheroscler. Thromb..

[9] Kubo H., Fujii K., Kawabe T., Matsumoto S., Kishida H., Hosoe K. (2008). Food content of ubiquinol-10 and ubiquinone-10 in the Japanese diet. J. Food Comp. Anal..

[10] Kishi T. (1982). Concentration of CoQ10 in serum and urine after intake. Iyaku Ja-naru.

[11] Yamagishi K., Ikeda A., Moriyama Y., Chei C.L., Noda H., Umesawa M., Cui R., Nagao M., Kitamura A., Yamamoto Y. (2014). Serum coenzyme Q10 and risk of disabling dementia: The circulatory risk in communities study (CIRCS). Atherosclerosis.

[12] Shimizu K., Kamei Y., Suzuki M., Eda N., Hanaoka Y., Kono I., Akama T. (2015). The effects of coenzyme Q10 on oral immunity and health-related quality of life in middle-aged and elderly individuals. Jpn. J. Complement. Alternat. Med..

[13] Kinoshita T., Fujii K. (2019). Long-term intake of ubiquinol may improve cognitive performance in community residents. J. Japanese Association Rural Med..

[14] Ikeda S., Toyoda K. (2009). The influence that CoQ10 gives psychological element. Seisen-ronnsou.

[15] Chalder T., Berelowitz G., Pawlikowska T., Watts L., Wessely S., Wright D., Wallace E.P. (1993). Development of a fatigue scale. J. Psychosom. Res..

[16] Fukuda S., Takashima S., Iwase M., Yamaguti K., Kuratsune H., Watanabe Y., Watanabe Y., Evengård B., Natelson B.H., Jason L.A., Kuratsune H. (2008). Development and validation of a new fatigue scale for fatigued subjects with and without chronic fatigue syndrome. Fatigue Science for Human Health.

[17] Kessler R.C., Andrews G., Colpe L.J., Hiripi E., Mroczek D.K., Normand S.-L.T., Walters E.E., Zaslavsky A.M. (2002). Short screening scales to monitor population prevalences and trends in non-specific psychological distress. Psychol. Med..

[18] Radloff L.S. (1977). The CES-D Scale: A self-report depression scale for research in the general population. Appl. Psychol. Meas..

[19] Buysse D.J., Reynolds C.F., Monk T.H., Berman S.R., Kupfer D.J. (1989). The Pittsburgh Sleep Quality Index: A new instrument for psychiatric practice and research. Psychiatry Res..

[20] Johns M.W. (1991). A new method for measuring daytime sleepiness: The Epworth sleepiness scale. Sleep.

[21] Doi Y., Minowa M., Uchiyama M., Okawa M., Kim K., Shibui K., Kamei Y. (2000). Psychometric assessment of subjective sleep quality using the Japanese version of the Pittsburgh Sleep Quality Index (PSQI-J) in psychiatric disordered and control subjects. Psychiatry Res..

[22] Furukawa T., Kawakami N., Saitoh M., Ono Y., Nakane Y., Nakamura Y., Tachimori H., Iwata N., Uda H., Nakane H. (2008). The performance of the Japanese version of the K6 and K10 in the World Mental Health Survey Japan. Int. J. Methods Psychiatr. Res..

[23] Shima S., Shikano T., Kitamura T., Asai M. (1985). New self-rating scales for depression. Clin. Psychiatry.

[24] Takegami M., Suzukamo Y., Wakita T., Noguchi H., Chin K., Kadotani H., Inoue Y., Oka Y., Nakamura T., Green J. (2009). Development of a Japanese version of the Epworth Sleepiness Scale (JESS) based on item response theory. Sleep Med..

[25] Tanaka M., Fukuda S., Mizuno K., Imai-Matsumura K., Jodoi T., Kawatani J., Takano M., Miike T., Tomoda A., Watanabe Y. (2008). Reliability and validity of the Japanese version of the Chalder Fatigue Scale among youth in Japan. Psychol. Rep..

[26] Kanaya N., Hirata N., Kurosawa S., Nakayama M., Namiki A. (2003). Differential effects of propofol and sevoflurane on heart rate variability. Anesthesiology.

[27] Takusagawa M., Komori S., Umetani K., Ishihara T., Sawanobori T., Kohno I., Sano S., Yin D., Ijiri H., Tamura K. (1999). Alterations of autonomic nervous activity in recurrence of variant angina. Heart.

[28] Akselrod S., Gordon D., Ubel F.A., Shannon D.C., Berger A.C., Cohen R.J. (1981). Power spectrum analysis of heart rate fluctuation: A quantitative probe of beat-to-beat cardiovascular control. Science.

[29] Pomeranz B., Macaulay R.J., Caudill M.A., Kutz I., Adam D., Gordon D., Kilborn K.M., Barger A.C., Shannon D.C., Cohen R.J. (1985). Assessment of autonomic function in humans by heart rate spectral analysis. Am. J. Physiol..

[30] Malliani A., Pagani M., Lombardi F., Cerutti S. (1991). Cardiovascular neural regulation explored in the frequency domain. Circulation.

[31] Appel M.L., Berger R.D., Saul J.P., Smith J.M., Cohen R.J. (1989). Beat to beat variability in cardiovascular variables: Noise or music?. J. Am. Coll. Cardiol..

[32] Mizuno K., Tanaka M., Yamaguti K., Kajimoto O., Kuratsune H., Watanabe Y. (2011). Mental fatigue caused by prolonged cognitive load associated with sympathetic hyperactivity. Behav. Brain Funct..

[33] Tajima K., Tanaka M., Mizuno K., Okada N., Rokushima K., Watanabe Y. (2008). Effects of bathing in micro-bubbles on recovery from moderate mental fatigue. Ergonomia IJE & HF.

[34] Mizuno K., Tanaka M., Tajima K., Okada N., Rokushima K., Watanabe Y. (2010). Effects of mild-stream bathing on recovery from mental fatigue. Med. Sci. Monit..

[35] Mizuno K., Sasaki A.T., Ebisu K., Tajima K., Kajimoto O., Nojima J., Kuratsune H., Hori H., Watanabe Y. (2018). Hydrogen-rich water for improvements of mood, anxiety, and autonomic nerve function in daily life. Med. Gas Res..

[36] Trotti R., Carratelli M., Barbieri M. (2002). Performance and clinical application of a new, fast method for the detection of hydroperoxides in serum. Panminerva Med..

[37] Nojima J., Motoki Y., Tsuneoka H., Kuratsune H., Matsui T., Yamamoto M., Yanagihara M., Hinoda Y., Ichihara K. (2011). ‘Oxidation stress index’ as a possible clinical marker for the evaluation of non-Hodgkin lymphoma. Br. J. Haematol..

[38] Kajimoto O., Watanabe Y., Evengård B., Natelson B.H., Jason L.A. (2008). Development of a method of evaluation of fatigue and its economic impacts. Fatigue Science for Human Health.

[39] Mizuno K., Tanaka M., Fukuda S., Imai-Matsumura K., Watanabe Y. (2011). Relationship between cognitive functions and prevalence of fatigue in elementary and junior high school students. Brain Dev..

[40] Mizuno K., Watanabe Y. (2013). Neurocognitive impairment in childhood chronic fatigue syndrome. Front. Physiol..

[41] Kawatani J., Mizuno K., Shiraishi S., Takao M., Joudoi T., Fukuda S., Watanabe Y., Tomoda A. (2011). Cognitive dysfunction and mental fatigue in childhood chronic fatigue syndrome--a 6-month follow-up study. Brain Dev..

[42] Mizuno K., Tanaka M., Fukuda S., Sasabe T., Imai-Matsumura K., Watanabe Y. (2011). Changes in cognitive functions of students in the transitional period from elementary school to junior high school. Brain Dev..

[43] Tanaka M., Shigihara Y., Funakura M., Kanai E., Watanabe Y. (2012). Fatigue-associated alterations of cognitive function and electroencephalographic power densities. PLoS ONE.

[44] Hart R.P., Kwentus J.A., Wade J.B., Hamer R.M. (1987). Digit symbol performance in mild dementia and depression. J. Consult. Clin. Psychol..

[45] Kertzman S., Nahum Z.B., Gotzlav I., Grinspan H., Birger M. (2006). Digid symbol substitution test performance: Sex differences in a Hebrew-readers’ health population. Percept. Mot. Skills.

[46] Lafont S., Marin-Lamellet C., Paire-Ficout L., Thomas-Antérion C., Laurent B., Fabrigoule C. (2010). The Wechsler digit symbol substitution test as the best indicator of the risk of impaired driving in Alzheimer disease and normal aging. Dement. Geriatr. Cogn. Disord..

[47] Ancoli-Israel S., Cole R., Alessi C., Chambers M., Moorcroft W., Pollak C.P. (2003). The role of actigraphy in the study of sleep and circadian rhythms. Sleep.

[48] Martin J.L., Hakim A.D. (2011). Wrist actigraphy. Chest.

[49] Kume S., Yamato M., Tamura Y., Jin G., Nakano M., Miyashige Y., Eguchi A., Ogata Y., Goda N., Iwai K. (2015). Potential biomarkers of fatigue identified by plasma metabolome analysis in rats. PLoS ONE.

[50] Singh A., Garg V., Gupta S., Kulkarni S.K. (2002). Role of antioxidants in chronic fatigue syndrome in mice. Indian J. Exp. Biol..

[51] Fukuda S., Nojima J., Motoki Y., Yamaguti K., Nakatomi Y., Okawa N., Fujiwara K., Watanabe Y., Kuratsune H. (2016). A potential biomarker for fatigue: Oxidative stress and anti-oxidative activity. Biol. Psychol..

[52] Miller R.W., Curry J.R. (1969). Mammalian dihydroorotate--ubiquinone reducatse complex. II. Correlation with cytochrome oxidase, mode of linkage with the cytochrome chain, and general properties. Can. J. Biochem..

[53] Takayanagi R., Takeshige K., Minakami S. (1980). NADH- and NADPH-dependent lipid peroxidation in bovine heart submitochondrial particles. Dependence on the rate of electron flow in the respiratory chain and an antioxidant role of ubiquinol. Biochem. J..

[54] Kagan V., Serbinova E., Packer L. (1990). Antioxidant effects of ubiquinones in microsomes and mitochondria are mediated by tocopherol recycling. Biochem. Biophys. Res. Commun..

[55] Mohr D., Bowry V.W., Stocker R. (1992). Dietary supplementation with coenzyme Q10 results in increased levels of ubiquinol-10 within circulating lipoproteins and increased resistance of human low-density lipoprotein to the initiation of lipid peroxidation. Biochem. Biophys. Acta.

[56] Stough C., Nankivell M., Camfield D.A., Perry N.L., Pipingas A., MacPherson H., Wesnes K., Ou R., Hare D., De Haan J. (2019). CoQ10 and cognition a review and study protocol for a 90-day randomized controlled trial investigating the cognitive effects of ubiquinol in the healthy elderly. Front. Aging Neurosci..

[57] Tajima S., Yamamoto S., Tanaka M., Kataoka Y., Iwase M., Yoshikawa E., Okada H., Onoe H., Tsukada H., Kuratsune H. (2010). Medial orbitofrontal cortex is associated with fatigue sensation. Neurol. Res. Int..

[58] Mizuno K., Tanaka M., Ishii A., Tanabe H.C., Onoe H., Sadato N., Watanabe Y. (2008). The neural basis of academic achievement motivation. Neuroimage.

[59] Critchley H.D., Mathias C.J., Josephs O., O’Doherty J., Zanini S., Dewar B., Cipolotti L., Shallice T., Dolan R. (2003). Human cingulate cortex and autonomic control: Converging neuroimaging and clinical evidence. Brain.

[60] Morris G., Anderson G., Maes M. (2017). Hypothalamic-Pituitary-Adrenal Hypofunction in Myalgic Encephalomyelitis (ME)/Chronic Fatigue Syndrome (CFS) as a Consequence of Activated Immune-Inflammatory and Oxidative and Nitrosative Pathways. Mol. Neurobiol..

[61] Watanabe K., Nozaki S., Goto M., Kaneko K.-I., Hayashinaka E., Irie S., Nishiyama A., Kasai K., Fujii K., Wada Y. (2019). PET imaging of ^11^C-labeled coenzyme Q10: Comparison of biodistribution between [^11^C]ubiquinol-10 and [^11^C]ubiquinone-10. Biochem. Biophys. Res. Commun..

[62] Yamano E., Sugimoto M., Hirayama A., Kume S., Yamato M., Jin G., Tajima S., Goda N., Iwai K., Fukuda S. (2016). Index markers of chronic fatigue syndrome with dysfunction of TCA and urea cycles. Sci. Rep..

